# Cryptogenic stroke and small fiber neuropathy of unknown etiology in patients with *alpha-galactosidase A* -10T genotype

**DOI:** 10.1186/s13023-014-0178-5

**Published:** 2014-11-26

**Authors:** Michael Schelleckes, Malte Lenders, Katrin Guske, Boris Schmitz, Christian Tanislav, Sonja Ständer, Dieter Metze, Istvan Katona, Joachim Weis, Stefan-Martin Brand, Thomas Duning, Eva Brand

**Affiliations:** Internal Medicine D, Department of Nephrology, Hypertension and Rheumatology, University Hospital Muenster, Albert-Schweitzer-Campus 1, 48149 Muenster, Germany; Institute of Sports Medicine, Molecular Genetics of Cardiovascular Disease, University Hospital Muenster, Horstmarer Landweg 39, 48149 Muenster, Germany; Department of Neurology, Justus Liebig University Giessen, Klinikstrasse 33, 35385 Giessen, Germany; Department of Dermatology, Neurodermatology and Competence Center Pruritus, University of Muenster, Von-Esmarch-Straße 58, 48149 Muenster, Germany; Institute of Neuropathology, University Hospital Aachen, Pauwelsstraße 30, 52074 Aachen, Germany; Department of Neurology, University Hospital Muenster, Albert-Schweitzer-Campus 1, 48149 Muenster, Germany

**Keywords:** Neuropathic pain, Stroke, Cerebrovascular disease, Fabry disease, Gene expression regulation

## Abstract

**Background:**

Fabry disease (FD) is a multisystemic disorder with typical neurological manifestations such as stroke and small fiber neuropathy (SFN), caused by mutations of the *alpha-galactosidase A* (*GLA*) gene. We analyzed 15 patients carrying the *GLA* haplotype -10C>T [rs2071225], IVS2-81_-77delCAGCC [rs5903184], IVS4-16A>G [rs2071397], and IVS6-22C>T [rs2071228] for potential neurological manifestations.

**Methods and results:**

Patients were retrospectively analyzed for stroke, transient ischemic attack (TIA), white matter lesions (WML) and SFN with neuropathic pain. Functional impact of the haplotype was determined by molecular genetic methods including real-time PCR, exon trapping, promoter deletion constructs and electrophoretic mobility shift assays. Symptomatic -10T allele carriers suffered from stroke, TIA, WML, and SFN with neuropathic pain. Patients’ mean GLA mRNA expression level was reduced to ~70% (p < 0.0001) and a dose-dependent effect of the -10T allele on GLA mRNA expression was observed in hemi/homozygous compared to heterozygous patients (p < 0.0001). Molecular analyzes revealed that the -10T allele resulted in a reduced promoter activity and an altered transcription factor binding, while a functional relevance of the co-segregated intronic variants was excluded by exon trapping.

**Conclusions:**

Based on this complementary approach of clinical observation and functional testing, we conclude that the *GLA* -10T allele could be causal for the observed neurological manifestations. Future studies are needed to clarify whether affected patients benefit from GLA enzyme replacement therapy for end-organ damage prevention.

**Electronic supplementary material:**

The online version of this article (doi:10.1186/s13023-014-0178-5) contains supplementary material, which is available to authorized users.

## Background

A deficiency of alpha-galactosidase A (GLA, E.C.3.2.1.22) leads to Fabry disease (FD), an X-linked lysosomal storage disorder. The impaired glycosphingolipid catabolism provokes progressive accumulation of glycosphingolipids, mainly globotriaosylceramide (Gb3), resulting in a multisystemic disease [1]. Progressive accumulation of Gb3 leads to macro- and microangiopathic alterations with transient ischemic attack (TIA), stroke, myocardial infarction, life-threatening cardiac arrhythmia and end-stage renal disease resulting in a 10–15 years reduced life-span without adequate therapy [2]. First symptoms of FD include neuropathic pain attacks due to small fiber neuropathy (SFN) [2,3]. FD routine diagnosis is based on the described typical clinical picture, decreased enzymatic GLA activities and analysis of coding *GLA* variants.

The non-coding *GLA* -10T allele (rs2071225), located within the 5′-untranslated region (UTR), has been suggested to be associated with decreased GLA protein expression [4], although the -10T allele co-segregates in a haplotype background with three additional intronic variants (IVS2­81_-77delCAGCC [rs5903184], IVS4-16A>G [rs2071397], and IVS6-22C>T [rs2071228]) [5,6]. This haplotype has been reported in patients with SFN of unknown etiology as well as in patients with classical FD [5,6]. Until now, the functional role of the -10T allele and the co-segregating intronic variants remains unclear [6]. In contrast to mutations in coding regions affecting peptide sequences and possibly modifying protein structure and function, the consequences of intronic sequences are not predictable. As shown for the mid-intronic *GLA* mutation IVS4+919A>G, intronic variations can affect the process of alternative splicing [7,8]. Due to this mutation, a weak splice site can be converted, resulting in an increased recognition and the insertion of an intronic sequence into the GLA transcript leading to a cardiac phenotype of FD [7,8].

In general, efficient splicing of pre-mRNAs depends on conserved intronic sequences. The efficiency of splicing can further be modified by splicing enhancers or suppressors, sequences located within exons and introns. As opposed to the well-defined consensus splice sites, these elements are not completely characterized. Thus, a prediction whether a genomic variation affects splicing is not possible yet and the impact has to be confirmed experimentally [9].

In the current work, we retrospectively analyzed 15 -10T allele carrying patients from our database after presentation of a symptomatic index patient with a neurological phenotype. Our complementary approach included clinical data and detailed molecular functional analyses.

## Methods

### Patients

**Figure 1:**
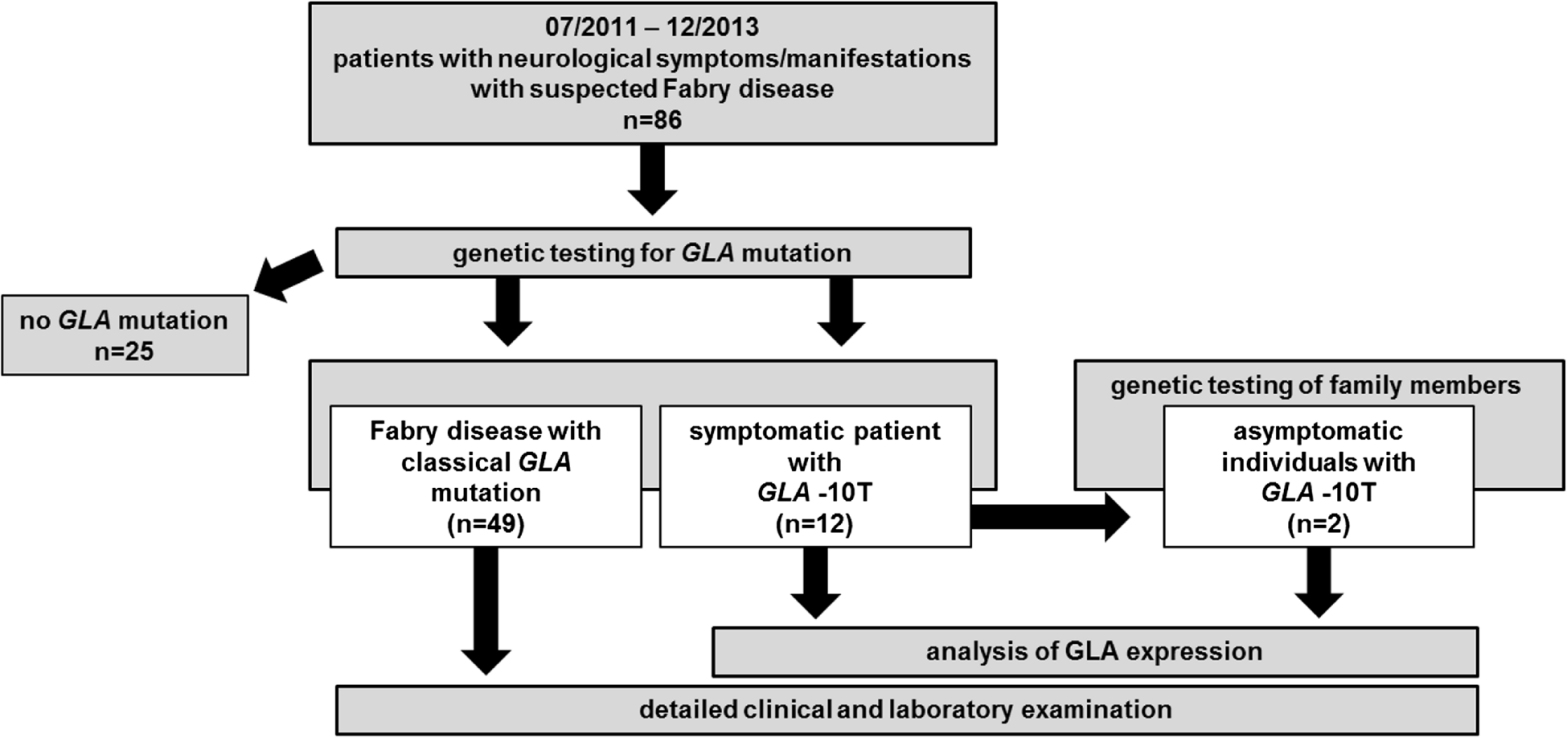
**Presentation of the retrospective study design.** Between 07/2011 and 12/2013 86 new patients presented at the Fabry center of the University Hospital Muenster with Fabry-typical neurological manifestations such as small fiber neuropathy with neuropathic pain, or stroke/TIA of unknown etiology. Out of 86 patients 49 had classical Fabry disease-causing mutations, 12 patients were symptomatic -10T allele carriers and 25 patients had no *GLA* mutation. Two asymptomatic patients were identified by family screening. We analyzed GLA expression in all detected -10T allele carriers and retrospectively analyzed clinical data.

The study retrospectively analyzed patients with the *GLA* -10T haplotype who presented at the Fabry center of the University Hospital of Muenster (IFAZ) between 07/2011 and 12/2013 (Figure 1). All patients had been examined by neurologists, cardiologists and nephrologists at the Fabry center. Neuropathic pain was diagnosed according to the revised criteria of the Neuropathic Pain Special Interest Group of the International Association for the Study of Pain (NeuPSIG) [10]. All investigations were performed after approval of the Medical Association of Westfalian Lippe and the Ethical Committee of the Medical Faculty of the University of Muenster (project-no.: 2011-347-f, date of report: 07.07.2011). Written informed consent of patients was obtained for molecular analysis and publication.

### GLA activity, lyso-Gb3 measurements and *GLA* sequencing

GLA activity was determined using 4-methylumbelliferyl-α-D-galactopyranoside (Santa Cruz Biotechnology, Heidelberg, Germany), as described previously [11]. N-acetylgalactosamine (Santa Cruz Biotechnology) was used as specific inhibitor of endogenous α-Galactosidase B activity [12]. GLA enzyme activity was determined as nanomoles (nmol) of substrate hydrolyzed per hour (h) per mg protein. For lyso-Gb3, lyso­Ceramide was used as reference (Matreya LLC, Pleasant Gap, USA) and D5-fluticasone propionate (EJY Tech, Inc., Rockville, USA) served as internal standard. Genomic DNA (isolated from leukocytes) had been sequenced for *GLA* exons and 30–50 bp of adjacent introns.

### Magnetic resonance imaging data analysis

Cerebral lesion volume on axial fluid attenuated inversion recovery sequences was determined semi-automatically by outlining the peripheral borders of white matter lesions (WML). Lesions were marked and borders were set by local thresholding using a custom-tailored software based on Analysis-software (Brain Imaging Resource, Mayo Clinic, Rochester, USA). WML were additionally rated on a 3-point scale according to the well-established score of Fazekas [13].

### Skin biopsy histology

A 3 mm skin punch biopsy was obtained from the right distal calf and fixed in Zamboni solution. Forty μm cryostat sections were stained with a polyclonal rabbit anti-PGP9.5 antiserum (Ultraclone, Yarmouth, UK) as described previously [14]. Immunoreactivity was visualized using Alexa Fluor 488 (Invitrogen, Darmstadt, Germany) labeled goat anti-rabbit secondary antibodies. Intra-epidermal nerve fiber density (IENFD) was determined using the method described by Lauria et al. [15], counting only nerve fibers crossing the epidermal basement membrane and excluding nerve fragments in the epidermis that did not cross the basement membrane. Specimen length was determined using the scale bar in the microscope eyepiece calibrated with a standardized scale bar slide. The normal value references were >9 fibers/mm based on published [15] and own control subjects (n = 66) normative data. In addition, axons showing focal swellings of >1.5 μm in diameter were counted. Such swellings are supposed to indicate disturbances in axonal transport [16].

### Quantitative sensory testing

All patients underwent the QST protocol as developed by the German Research Network on Neuropathic Pain [17,18]. This protocol encompasses the following items: cold detection threshold (CDT), warm detection threshold (WDT), cold pain threshold (CPT), heat pain threshold (HPT), thermal sensory limen (TSL), the presence of paradoxical heat sensations (PHS), mechanical detection thresholds (MDT) to von Frey filaments, vibration sensation (VDT) making use of a 64-Hz tuning fork, mechanical pain thresholds to pinprick stimuli (MPT) and blunt pressure (PPT), stimulus–response–functions for pinprick (MPS) and dynamic mechanical allodynia (DMA), and pain summation (wind-up ratio, WUR) using repetitive pinprick stimulation. The tests were all performed at the dorsum of the left hand and the dorsum of the right foot. The thermal tests were performed using a TSA 2001-II (Medoc, Israel). For individual analysis, each patient’s values were compared with published reference values [17]. Based on the log transformed raw values for each QST item, a Z-score sensory profile was calculated as follows: Z-score = (value of the subject – mean value of controls)/standard deviation of controls. Negative Z-scores indicate loss of sensation, positive Z-scores indicate gain of sensation.

### Transmission electron microscopy

For electron microscopy specimens were fixed in Karnovsky’s fixative, postfixed in 1% osmium tetroxide and embedded in Epon. Ultrathin sections were counterstained with uranyl acetate and lead citrate and examined in a Philips CM10 electron microscope.

### Real-time PCR analysis

Total RNA extraction from patients’ peripheral mononuclear blood cells was performed using the NucleoSpin RNA Blood Kit (Macherey-Nagel, Düren, Germany). Five hundred ng of patients’ total RNA was reverse transcribed using SuperScriptII Reverse Transcriptase (Invitrogen). Relative GLA transcript levels (NM_000169.2) were analyzed in duplicates using Power SYBR Green (Applied Biosystem, Carlsbad, USA) on an Applied Biosystems 7500 Fast real-time PCR system. Glyceraldehyde 3-phosphate dehydrogenase was used as endogenous reference control. Oligonucleotides used for amplification are given in Additional file 1: Table S2. Amplification standard curves were generated by cDNA serial dilutions. Melting curve analysis was used to assess PCR specificity. Relative expression level of GLA was analyzed using the 2^−ΔΔCt^ method [19]. The absence of non-specific amplification was confirmed by agarose gel electrophoresis of PCR amplicons. Real-time PCR has been repeated at least twice.

### Cell culture

Immortalized human kidney epithelial cells (IHKE) [20–22] were maintained in Dulbecco’s modified Eagle’s medium/Ham’s-F12 with 1% fetal calf serum (FCS; PAA, Cölbe, Germany), 100 units/ml penicillin, 100 ng/ml streptomycin, 2 mmol/ml L-glutamine, 10 ml/l insulin-transferrin-sodium selenite media supplement, 1.25 g/l NaHCO_3_, 55 mg/l sodium pyruvate, 10 μg/l human epidermal growth factor (all Sigma-Aldrich, Munich, Germany) and 15 mmol/l N-2-hydroxyethylpiperazine-N-2-ethanesulfonic acid (Merck, Darmstadt, Germany). The human vascular endothelial cell line EA.hy926 was maintained in Dulbecco’s modified Eagle’s medium with 10% FCS, 100 units/ml penicillin, 100 ng/ml streptomycin and 2 mmol/ml L-glutamine. The monocytic cell line THP-1 was maintained in RPMI 1640, 10% FCS, 100 units/ml penicillin, 100 μg/ml streptomycin, and 1x modified Eagle’s medium amino acid solution (Sigma-Aldrich). Human SH-SY5Y neuroblastoma cells were maintained in Dulbecco’s modified Eagle’s medium with 20% FCS, 100 units/ml penicillin, 100 ng/ml streptomycin and 2 mmol/ml L-glutamine. Cells were transfected using Nanofectin (PAA). The pGL3-Control vector, in which transcription is driven by a viral SV40-promoter, served as control for transfection efficiency. For transcription factor EB (TFEB) co-transfection assays, expression vector pcDNA3.1(+) TFEB and *GLA* promoter deletion constructs were transfected in a 3:1 ratio. Transfection experiments were repeated at least twice.

### Exon trapping assay

The influence of intronic *GLA* variants on splice events was analyzed by use of the exon trapping vector pSPL3. *GLA* exon 3 (IVS2-81_-77delCAGCC [rs5903184]), exon 5 (IVS4-16A>G [rs2071397]) and exon 7 (IVS6-22C>T [rs2071228]) were PCR-amplified with approximately 250 bp flanking introns (wild-type and mutation), and inserted into the pSPL3 vector. The intronic *GLA* variant IVS4+919G>A served as a positive control [7,23]. Minigene constructs were transfected into EA.hy926 and SH-SY5Y cells using Nanofectin (PAA). Twenty-four hours after transfection, RNA was extracted using NucleoSpin RNAII (Machery-Nagel), and single-stranded cDNA was synthesized (SuperScriptII Reverse Transcriptase, Invitrogen). PCR was performed using vector-specific primers SD6 and SA2. Signals were analyzed on 2% agarose gels. Identity of PCR amplicons was validated by direct sequencing. Oligonucleotide sequences are given in Additional file 1: Table S2.

### Serial promoter deletion constructs

*GLA* serial promoter deletion constructs were generated using the pGL3 reporter gene vector system (Promega, Mannheim, Germany) based on the *GLA* translational start site (NM_000169.2). Luciferase activities were determined using the luciferase assay kit (Promega) and a Sirius luminometer (Berthold detection systems, Pforzheim, Germany). The -10T allele was introduced by site-directed mutagenesis. All vectors were sequenced to ensure sequence accuracy and identity. Oligonucleotide sequences are given in Additional file 1: Table S2.

### Chromatin immunoprecipitation (ChIP)

ChIP was performed as described previously [24,25]. A Bioruptor (Diagenode, Liège, Belgium) was used for DNA sonication until the chromatin had an average size of 300–500 bp (≤45 min, 0.5 s interval, 200 W, 4°C). ChIP was conducted using 3 μg of TFEB antibody (TFEB-C6, #166736, Santa Cruz Biotechnology). ChIP experiments were repeated twice. Oligonucleotides used for amplification of precipitated chromatin are given in Additional file 1: Table S2.

### Electrophoretic mobility shift assay (EMSA)

Nuclear protein extracts of EA.hy926 cells were prepared by a modified protocol [26]. Oligonucleotides for DNA probes containing position -10 of the *GLA* 5′-UTR were 3′-biotinylated with biotin-16-ddUTP (Roche, Mannheim, Germany) using terminal transferase (Roche). Per reaction, 5 μg nuclear protein extracts were incubated with 500 ng pre-sheared poly(dI•dC) (USB, Staufen, Germany) as non-specific competitor and a 200-fold molar excess of unlabeled oligonucleotides as specific competitor. The EMSA shown is representative for at least two experiments. Oligonucleotide sequences are given in Additional file 1: Table S2.

### Statistical analysis

Two-tailed student’s *t*-test was used for statistical analysis. P-values <0.05 were considered as statistically significant.

## Results

### Symptomatic carriers of the minor -10T allele suffer from neurological manifestations and show decreased GLA mRNA expression levels

**Figure 2:**
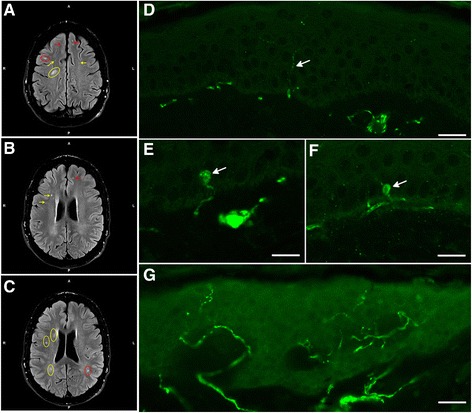
**Magnetic resonance imaging (MRI) and small fiber neuropathy diagnosis by PGP9.5 immunofluorescence. (A-C)** Fluid attenuated inversion recovery (FLAIR) MRI of patient #1 showed multiple, punctuated white matter lesions from periventricular (yellow arrows and circles) to subcortical (red arrows and circles) without gadolinium enhancement. **(D)** Skin biopsy analysis of patient #2 showed the reduction of the small epidermal nerve fibers (arrow). Scale bar = 20 μm. **(E-F)**. The number of focal axonal swellings (arrows) larger than 1.5 μm was increased. Scale bar = 10 μm. **(G)** Skin biopsy of a healthy control. Scale bar = 30 μm.

An index patient (patient #1) presented at the Fabry center of the University Hospital of Muenster (IFAZ) with severely impaired daily activity and reduced quality of life due to neuropathic pain of the distal extremities with “burning” hands and feet and physical weakness at the age of 56 years (Tables 1 and 2, Additional file 1: Table S1). She reported about first neuropathic pain symptoms at the age of 40 years. In-depths clinical and laboratory investigations revealed a SFN and widespread periventricular to subcortical WML (Figure 2A-C). Left-ventricular hypertrophy and diastolic ventricular dysfunction indicated cardiac involvement. A detailed laboratory investigation and cerebrospinal fluid (CSF) analysis were unrevealing. The patient had an unrevealing cardiovascular risk profile and no evidence of extracerebral arteriopathy, vasculitis or other inflammatory disease. Since further investigations revealed no evidence for common causes of SFN, the patient was tested for FD. Direct *GLA* sequencing revealed -10T heterozygosity in the 5′-UTR in a haplotype with the three intronic variants (IVS2-81_-77delCAGCC, IVS4-16A>G and IVS6-22C>T). No other mutation within the *GLA* coding region or exon-intron boundaries was found (Table 1, Additional file 1: Table S1). Since several reports existed on -10T heterozygosity and Fabry-typical manifestations [4–6] we retrospectively analyzed additional 14 -10T allele carriers (44.4 [12–72] years of age) from our database (Figure 1, Tables 1 and 2, Additional file 1: Table S1). Out of 15 -10T carriers 13 presented with neuropathic pain (symptom onset: patients #3-7 and #9-11 in childhood; #2 at 20 and #8 at 40 years of age) and/or cerebrovascular manifestations such as WML, TIA or stroke (Table 1). Blood count (including total leukocyte count), renal and liver function tests as well as homocysteine level and angiotensin-converting enzyme were normal. Serum antibodies against thyroid peroxidase, thyreoglobulin, glutamic acid decarboxylase, and onconeural antibodies (anti-amphiphysin, anti-Ri, anti-Yo, anti-Hu, anti-CV2/CRMP5, anti-Ma2/Ta, anti-NMDA, LGI-1, GAD) as well as anti-cardiolipin immunoglobulin were all negative. A screen for antibodies against extractable nuclear and anti-nuclear antigens was also negative. No patient had evidence of an inflammatory disease since serological testing excluded borreliosis, syphilis and HIV-1 infection. Cerebral MR-angiography was obtained in all patients with WML and showed no evidence of vasculitis. Sonography of the extracranial vessels were performed in all patients with stroke/TIA or WML (patients #1; #7; #8; #10-13) and showed no severe arteriopathy. Further examination revealed no evidence for common causes of SFN since blood glucose and hemoglobin A1c levels, blood count including total leukocyte count, c-reactive protein, thyroid and liver parameters, vitamins B1, B6, B12 and homocysteine levels, serum protein and immunoelectrophoresis were normal. CSF analyses were obtained in patients #1, #2, #8, and #11-13 and showed unrevealing results. Since CSF analyses were not performed in all patients, we could not definitively exclude inflammatory disease. However, no patient fulfilled the diagnostic criteria of primary vasculitis of the CNS or multiple sclerosis, regardless of CSF analysis results. Furthermore, the course of the disease and the additional diagnostic results did not agree with this diagnosis. All patients who presented with WML or stroke/TIA were non-smokers. Except for patients #8 and #12 who presented with well-controlled arterial hypertension, the remaining patients with stroke/TIA or WML had normal blood pressure. Only patient #6 was consecutively diagnosed with type 2 diabetes mellitus. Fabry-typical manifestations such as hypohidrosis, angiokeratoma, severe gastrointestinal disturbances and tinnitus were also observed (Additional file 1: Table S1), while only two patients presented renal manifestations.

**Table 1:** **Patients’ characteristics**

				**Neurological manifestations**							**WML characterization**		
**Patient #**	**m/f**	**Age [years]**	**Genotype**	**General**	**Neuropathic pain** ^**+**^	**NRS (most severe pain)**	**NRS (mean pain)**	**IENFD (anti-PGP9.5) [fibers/mm]**	**QST**	**Cerebrovascular manifestations [age of onset, years]**	**Localization**	**FS**	**V [ml]**
**-10T carriers with neurological manifestations**													
1*	f	63	-10T/X	Neuropathic pain, allodynia, autonomic neuropathy (postprandial gastrointestinal dysfunction)	Definite	Before ERT: 10	Before ERT: 8	4.64^†^	Abnormal	WML, TIA [62]	Periventricular and subcortical	1	10.9
						After ERT: 4	After ERT: 2						
2	f	43	-10T/X	Neuropathic pain, allodynia	Definite	7	4	4.4^†^	Abnormal	-	-	-	-
3	m	12	-10T/Y	Neuropathic pain, chronic headache	Definite	4	1	NA	Abnormal	-	-	-	-
4	m	15	-10T/Y	Neuropathic pain, autonomic neuropathy (postprandial gastrointestinal dysfunction)	Definite	6	3	NA	Abnormal	-	-	-	-
5	f	46	-10T/X	Neuropathic pain	Probable	6	5	NA	NA	-	-	-	-
6	f	53	-10T/X	Neuropathic pain	Probable	7	6	NA	Normal	-	-	-	-
7	f	65	-10T/X	Neuropathic pain, allodynia	Definite	9	5	4.65^†^	Abnormal	TIA [64]	-	-	-
8	f	49	-10T/X	Neuropathic pain, chronic headache	Possible	8	6	NA	NA	WML, TIA [48]	Periventricular and subcortical, confluent	2	17.8
9	f	55	-10T/X	Neuropathic pain	Probable	10	8	13.4	Abnormal	-	-	-	-
10	f	54	-10T/-10T	Neuropathic pain	Definite	9	6	NA	Abnormal	TIA [52]	-	-	-
11	f	72	-10T/X	Neuropathic pain	Definite	3	5	3.7^†^	Abnormal	WML	Periventricular and subcortical, confluent	2	22.6
12	m	52	-10T/Y	None	No pain	0	0	NA	NA	Stroke [48]	-	-	-
13	m	35	-10T/Y	None	No pain	0	0	NA	NA	Stroke [34]	-	-	-
**-10T carriers without neurological manifestations**													
14	m	19	-10T/Y	none	No pain	0	0	NA	Normal	-	-	-	-
15	m	33	-10T/Y	none	No pain	0	0	NA	NA	-	-	-	-

^†^reduced intra-epidermal nerve fiber density (IENFD; <9 fibers/mm); f: female; m: male; WML: white matter lesions; TIA: transient ischemic attack; QST: quantitative sensory testing; NA: not available; ^+^: according to the Neuropathic Pain Special Interest Group (NeuPSIG); NRS: numeric rating scale (0–10 scale; 0: no pain, 10: worst possible pain); FS: Fazekas score: 0: no WML, 1: punctate foci of WML, 2: beginning confluence of foci of WML, and 3: large confluent areas of WML; V: volume; FD: Fabry disease; *: index patient.

**Table 2:** **Laboratory FD parameters**

**Patient #**	**Normal GLA activity [FI]**	**GLA mRNA expression [FI]**	**Lyso-Gb3 [ng/ml]**
1*	2.67	0.88	0.68
2	3.09	0.87	1.08
3	0.91^+^	0.57	0.46
4	1.76	0.52	0.63
5	2.12	0.69	0.20
6	2.00	NA	0.90
7	2.20	1.20	0.50
8	1.60	0.76	0.50
9	2.64	1.00	0.50
10	1.92	0.63	0.30
11	0.94^+^	0.85	0.60
12	0.61^+^	0.48	0.90
13	0.88^+^	NA	0.60
14	1.48	0.57	1.12
15	2.84	0.66	0.40

Reference values: GLA activity in leukocytes >33 nmol MU/h/mg protein; GLA activity in dried blood spots >2.5 μmol/l/h. Lyso-Gb3 plasma <2.2 ng/ml; NA: not available; FI: fold induction; FD: Fabry disease. *: index patient; ^+^: reduced GLA activity.

**Figure 3:**
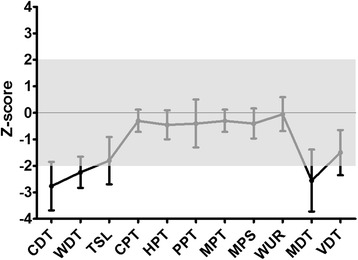
**Z-score sensory profiles of -10T allele carriers.** The Z-score profile of -10T allele carriers shows a predominant loss of sensory function in terms of cold and warm hypoesthesia (CDT, WDT, and TSL) and a reduced mechanical and vibratory sensation (MDT and VDT). The profile is consistent with a selective small fiber neuropathy in FD. Negative Z-scores indicate loss of sensation, positive Z-scores indicate gain of sensation. Error bars represent standard deviation of the mean; n = 10. CDT: cold detection threshold, WDT: warm detection threshold, TSL: thermal sensory limen, CPT: cold pain threshold, HPT: heat pain threshold, PPT: pressure pain threshold, MPT: mechanical pain threshold, MPS: mechanical pain sensitivity, WUR: wind-up ratio, MDT: mechanical detection threshold, VDT: vibration detection threshold.

**Figure 4:**
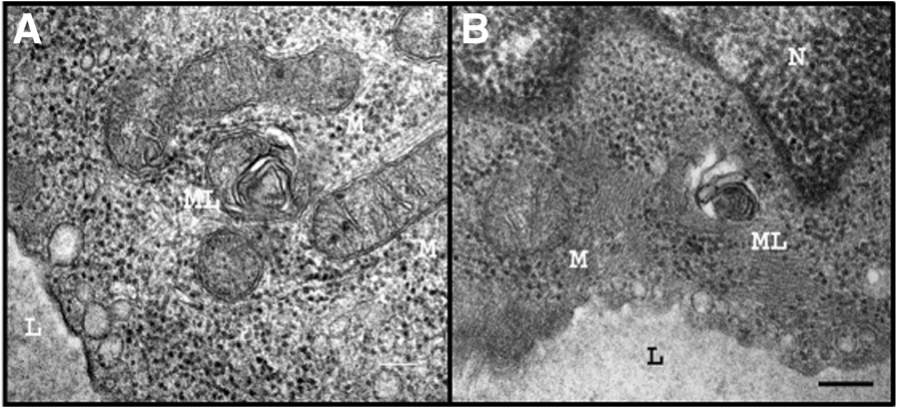
**Transmission electron microscopy (TEM) of skin biopsy of patient #6 (A) and #7 (B).** TEM of skin biopsies showed some endothelia of capillaries with irregular lamellar inclusions (ML). Mitochondria (M). Nucleus (N). Lumen of the capillary (L). Scale bar = 500 nm.

**Figure 5:**
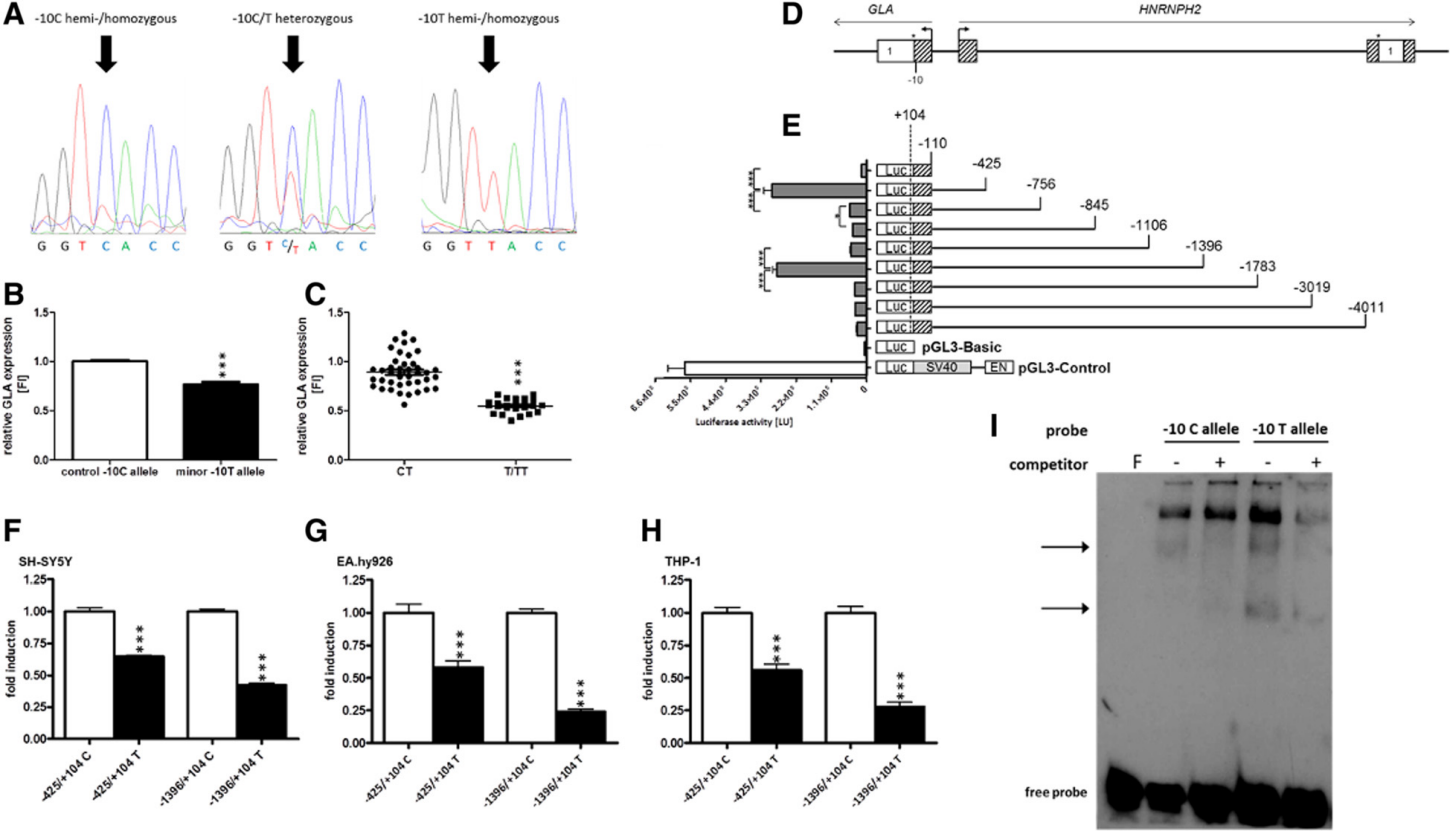
**Functional analysis of the -10T allele. (A)** Representative chromatograms showing nucleotide substitution at position -10. **(B)** Carriers of the minor -10T allele with neurological manifestations (black box; n = 11) showed significantly decreased GLA mRNA expression levels in peripheral mononuclear blood cells compared to healthy non-carriers (white box; male: n = 8; female: n = 9). **(C)** GLA expression is significantly decreased in symptomatic homo-/hemizygous T allele carriers (n = 4) versus symptomatic CT carriers (n = 7). **(D)** Schematic representation of the *GLA* 5′-flanking region. **(E)** Transient transfection of *GLA* promoter constructs in EA.hy926 cells revealed two regions with significant transcriptional activity. **(F-H)** Insertion of the minor T allele into promoter constructs (black bars) resulted in a decreased transcriptional activity in SH-SY5Y **(F)**, EA.hy926 **(G)** and THP-1 **(H)** cells. **(I)** EMSA with nuclear extract from EA.hy926 cells revealed one specific competable (non-allelic) band (upper arrow) and a slower migrating T allele-specific band (lower arrow). F: probe without extract and competitor. Data are given as mean ± SEM; LU: light units; Luc: *luciferase*.*p < 0.05; ***p < 0.001.

In detail, two patients (#12 and #13) suffered from stroke and three patients (#7, #8, and #10) from TIA (Table 1). In addition to the index patient, skin biopsies of three additional patients (#2, #7, and #11) revealed SFN diagnosed by reduction of IENFD (#1: 4.64/mm, #2: 4.40/mm, #7: 4.65/mm, #11: 3.70/mm; Figure 2D,G), while the number of focal axonal swelling of intra-epidermal axons was increased by 20% in patient #2 (Figure 2E,F). To evaluate the somatosensory phenotype of pain, QST was performed and was abnormal in 8 patients (#1-4, #7, and #9-11; Table 1), indicating functional impairment of A-delta- and C-fibers. In patients #1, #2, #4 and #7 DMA was present, and patients #3, #7, #9 and #10 had PHS. The Z-score profile of -10T allele carriers showed a predominant loss of sensory function in terms of cold and warm hypoesthesia (CDT, WDT, and TSL) and a reduced mechanical and vibratory sensation (MDT and VDT), a profile consistent with a selective small fiber neuropathy in FD (Figure 3) [27]. Available transmission electron microscopy of skin biopsies from patients #6 and #7 showed irregular, only partially concentric lamellar lysosomal inclusions devoid of strictly parallel membranes (Figure 4). Since the -10T haplotype may result in decreased mRNA expression we performed real-time PCR analysis revealing decreased mRNA expression levels in minor T allele carriers. This haplotype resulted in a significant GLA mRNA expression decrease of 30% compared to healthy controls (n = 17; p < 0.0001; Figure 5B). The observed mRNA reduction was associated with reduced enzymatic GLA activity in three patients (#3, #11 and #12; Table 2).

### Effect of -10T on GLA mRNA expression

**Figure 6:**
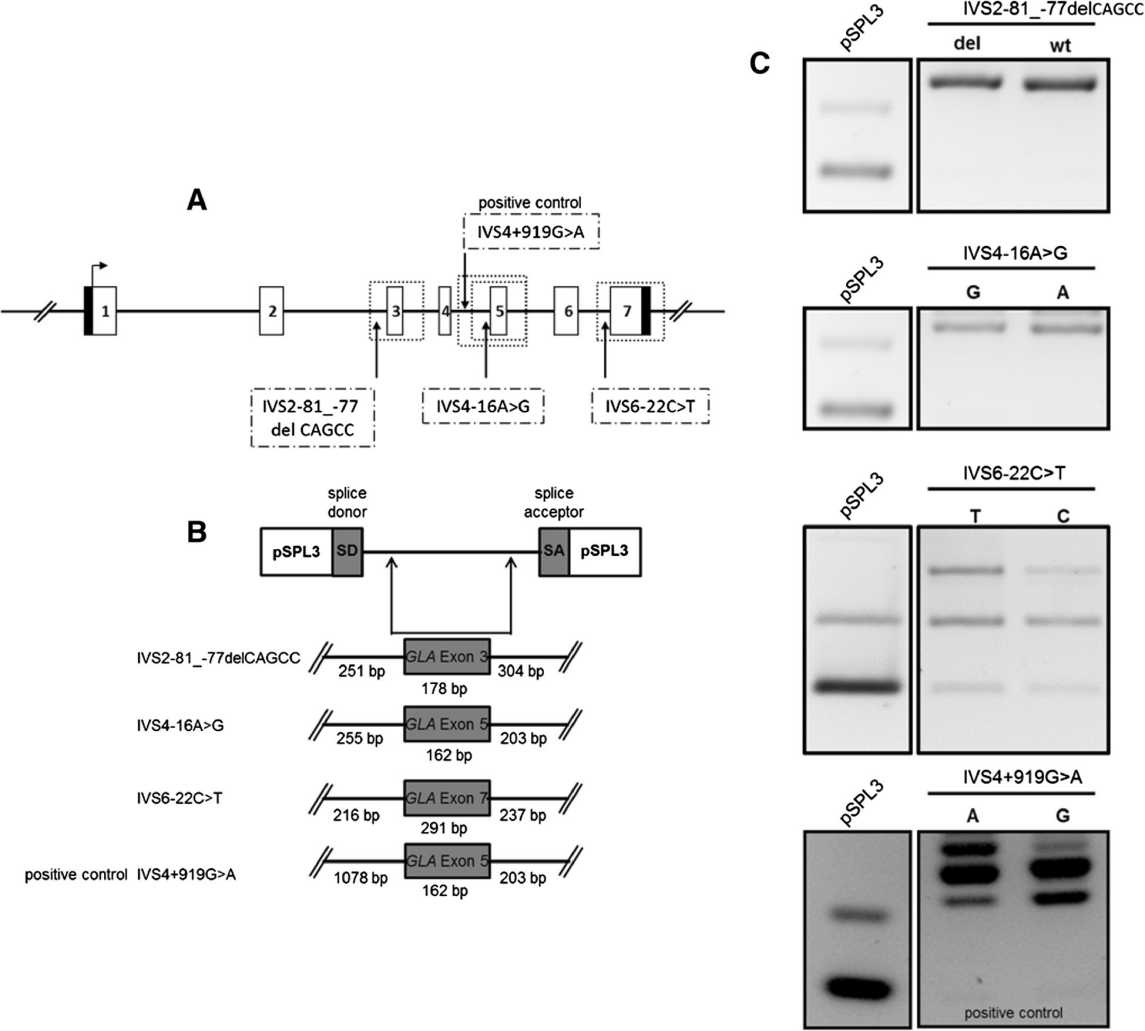
**Influence of intronic variants on GLA processing. (A)** Localization of the intronic variants IVS2-81_-77delCAGCC [rs5903184]), IVS4-16A>G [rs2071397] and IVS6-22C>T [rs2071228]). **(B)** *GLA* exons and denoted flanking introns (wild-type and variant). **(C)** Agarose gel separation of exon trapping products. wt: wild-type.

As intronic *GLA* variants such as IVS4+919G>A have been reported to cause late-onset FD with cardiac phenotype by altering GLA mRNA processing [7,23], we analyzed the potential effect of the intronic variants (IVS2-81_-77delCAGCC; IVS4-16A>G and IVS6-22C>T) co-segregating with the -10T allele by exon trapping experiments in an endothelial cell line (Figure 6). For all investigated intronic variants neither differences in transcript length nor sequence were detected. These observations suggest that the intronic variants IVS2-81_-77, IVS4-16 and IVS6-22 have no effect on GLA mRNA processing in contrast to the positive control IVS4+919G>A (Figure 6C). These results were confirmed in a neuronal cell line (data not shown). After exclusion of the functional impact of the intronic variants the reduction of GLA mRNA expression should be assigned to the -10T promoter allele.

### Identification of *GLA* regulatory promoter regions

Due to the localization of the -10T allele within the *GLA* 5′-UTR, we investigated *GLA* promoter transcriptional activity to identify *cis*-active regulatory elements. We generated serial promoter deletion constructs comprising up to 4,000 bp (Figure 5D). Transcriptional activity was assessed in the human endothelial cell line EA.hy926. Reporter gene assays revealed two regions in the *GLA* 5′-flanking region with strong transcriptional activity. A proximal region was located between positions -110 and -425 and a distal region between -1106 and -1396 (Figure 5E). The region between -1106 and -425 as well as -4011 and -1396 displayed only moderate transcriptional activity. Similar results were observed in IHKE (kidney epithelial), SH-SY5Y (neuroblastoma) and THP-1 (monocytic) cell lines (data not shown).

### Functional impact of the -10T allele

To investigate the functional impact of the -10T allele on *GLA* promoter activity, the -10T allele was introduced into the identified transcriptionally active promoter deletion constructs -425/+104 and -1396/+104 by site-directed mutagenesis (Figure 5F-H). Within the background of constructs −425/+104 and −1396/+104 the -10T allele insertion resulted in a significantly up to 3-fold decreased transcriptional activity (p < 0.001) in EA.hy926, SH-SY5Y and THP-1 cells. Similar results were observed in IHKE cells (data not shown).

EMSA experiments with probes resembling the region containing *GLA* position −10 resulted in specific binding patterns with nuclear proteins from EA.hy926 cells. We identified two specific band shifts, one of which was T allele-specific (Figure 5I), indicating altered transcription factor binding in the presence of the -10T allele.

To analyze whether this observation translates into a dose-dependent reduction of GLA mRNA expression, we compared hemi/homozygous with heterozygous patients carrying the ­10T allele. Confirming our previous observations, a significantly reduced GLA mRNA level was observed in hemi/homozygous patients, indicating a dose-dependent functional effect of the -10T allele (p < 0.0001; Figure 5C).

### Identification of transcription factor EB (TFEB) as a regulator of GLA expression

**Figure 7:**
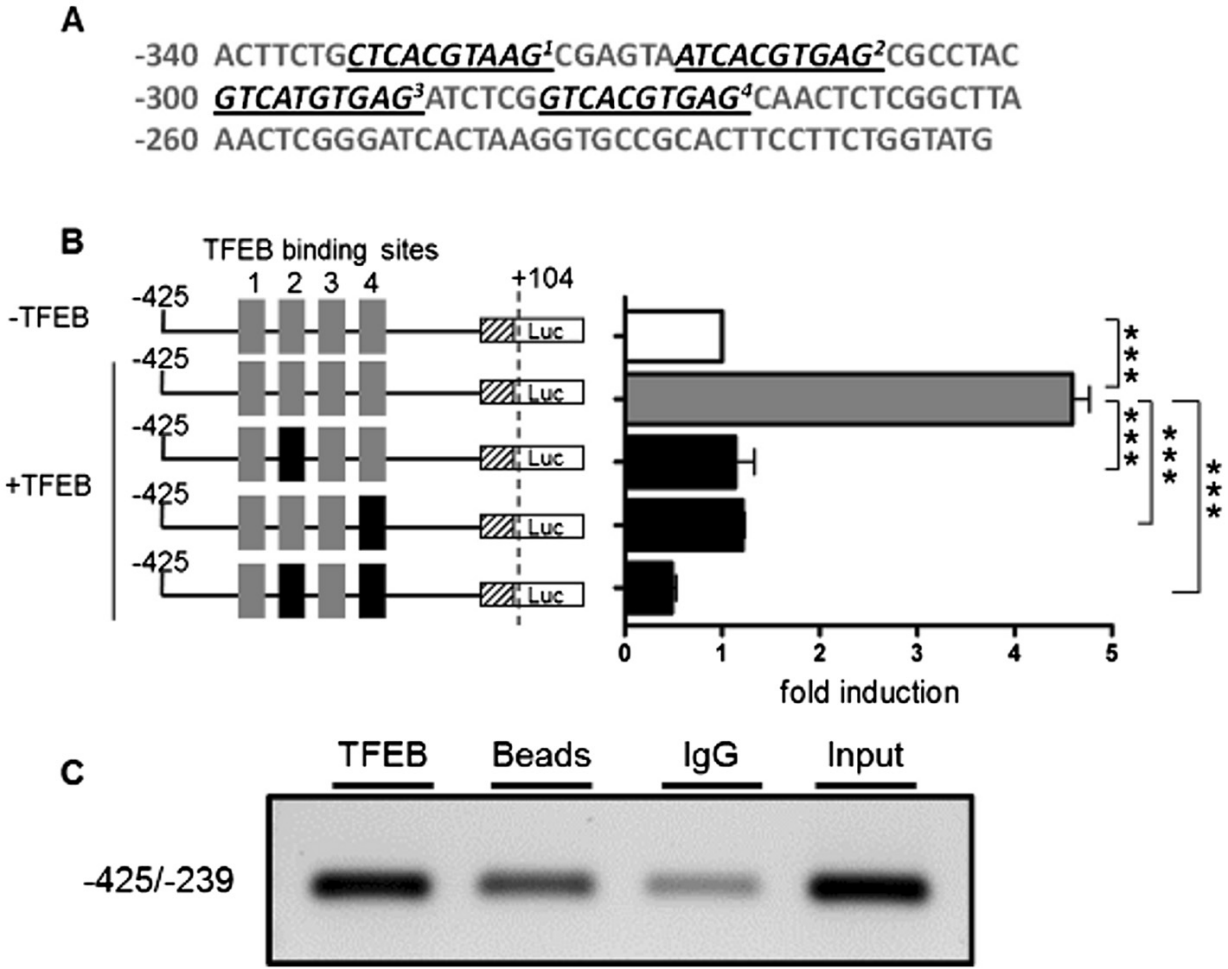
***GLA*** **promoter constructs are selectively activated by TFEB. (A)** Representation of the four putative TFEB binding sites (underlined) in the *GLA* promoter. **(B)**. Overexpression of TFEB in EA.hy926 cells (black bar) compared to mock transfected cells (white bar) and mutagenesis of conserved TFEB binding sites. **(C)** ChIP analysis in IHKE cells demonstrated the binding of TFEB. Input: Extracted chromatin served as positive control for PCR. Data are given as mean ± SEM. LU: light units; *Luc*: *luciferase*; ***p < 0.001.

To determine general regulatory factors involved in GLA expression regulation, we performed *in silico* analysis. We identified four putative TFEB binding sites within positions -333 to -274 from the translational start site (Figure 7A). Interestingly, Sardiello et al. [28] described TFEB as a “master regulator” of lysosomal genes. Subsequent overexpression of TFEB and *GLA* serial promoter deletion constructs in EA.hy926 cells resulted in a significant up to 4.5-fold increased (p < 0.001) transcriptional activity compared to the vector shuttle control (Figure 7B). Consequently, TFEB binding site mutation of two highly conserved binding sites led to total impairment of TFEB promoter activation (Figure 7B). Since this indicated a direct interaction of TFEB with the *GLA* promoter, we conducted ChIP experiments, which confirmed a specific interaction of TFEB with region -425 to -239 of the *GLA* promoter (Figure 7C).

## Discussion

Patients suffering from Fabry-typical manifestations without variants in the coding region represent a diagnostic/therapeutic dilemma as they are usually not treated with enzyme replacement therapy (ERT). In the current study, we describe a patient cohort suffering from neurological manifestations carrying the *GLA* -10T haplotype, which has been suggested to be associated with FD in family studies and case reports [4–6]. To the best of our knowledge, the molecular functional basis of the -10T haplotype has not been analyzed yet.

Our main findings are: 1) Symptomatic carriers of the -10T haplotype suffer from neurological manifestations and show decreased GLA mRNA expression levels. 2) Intronic variants transmitted together with -10T have no impact on GLA mRNA splicing. 3) The -10T allele is functionally relevant, leading to a significant decrease of *GLA* transcriptional activity as well as T allele-specific protein-DNA binding. 4) *GLA* gene expression depends on the lysosomal “master regulator” TFEB.

Thirteen patients with FD-typical neurological manifestations such as stroke, TIA, WML and SFN with neuropathic pain presenting at our Fabry center, had no classical FD-typical genetic variants, but the -10T haplotype. Recently, Pisani et al. [5] described a classical FD phenotype in a young female patient suffering from TIA associated with the -10T haplotype. In addition, Oliveira et al. [4] reported that the -10T haplotype was associated with cryptogenic cerebrovascular small-vessel disease. This genetic combination was also observed with high frequency (16%) in patients with SFN of unknown etiology [6]. These individual reports support our findings that the -10T haplotype might be associated with neurological manifestations.

Due to the retrospective study design skin biopsies, cerebral MRI and QST were not available for all patients of our study cohort. Skin biopsies of four patients out of five revealed SFN, with typical reduction of IENFD and focal axonal swelling of intra-epidermal axons. QST indicated functional impairment of A-delta- and C-fibers in 80% of the examined patients. All patients diagnosed with SFN by skin biopsy were also positive in QST analyses and further examination revealed no evidence for SFN of other etiologies. Transmission electron microscopy of two patients (#6 and #7) revealed lysosomal inclusions representing myelin-like figures, which could be precursors of FD-pathognomonic zebra bodies. A high proportion of the patients had reduced GLA mRNA expression levels, which were associated with reduced GLA enzyme activity in three patients, two hemizygous males and one 72 year old female patient. Notably, two of these patients presented WML or stroke as cerebrovascular manifestation. After exclusion of the functional impact of the co-segregated intronic variants, the observed neurological manifestations, which might be based on GLA mRNA expression reduction, may depend on the identified functional impact of the -10T promoter allele.

Our analysis confirms that the presence of the -10T allele caused altered transcription factor binding and reduced *GLA* promoter transcriptional activity as a potential cause for the observed reduction of GLA expression in our patients. Of note, the -10T allele showed a dose-dependent reduction of GLA expression in our patients. The observed mRNA reduction translated into reduced enzymatic GLA activity in three patients when compared to the reference values and hemi-/homozygous carriers of the minor T allele in our cohort tended to have decreased enzymatic activities compared to heterozygous T allele carriers. Consistently, previous studies on -10T allele carriers also reported slightly reduced or normal GLA activities in plasma or leucocytes [5,6]. Considering the observed clinical picture of our patients, it seems conceivable that neurological manifestations may occur in certain, mainly hemi- or homozygous -10T individuals, while GLA activities in plasma or leucocytes are still in the reference range.

Of note, plasma lyso-Gb3 levels of our patients were within the reference range. Lyso-Gb3 is proposed as a potential biomarker for classical FD phenotypes. The level of plasma lyso-Gb3 in affected patients is often higher than in mildly-affected patients [29,30]. Notably, it has been emphasized that atypical FD variants are often not associated with increased lyso-Gb3 levels, although biopsies of affected organs revealed lamellar inclusion bodies characteristic for FD [31,32]. A FD screening program in young patients with cryptogenic ischemic stroke identified a patient with the -10T haplotype [33]. The plasma lyso-Gb3 level of this patient was moderately increased in the first measurement and normal at retest, suggesting variability of the lyso-Gb3 concentration. These observations suggest that lyso-Gb3 is of limited use in FD variants.

The neurological system of FD patients is highly sensitive to reduced GLA enzyme activity, leading to neuropathic pain as one of the first symptoms in childhood [34,35]. The potential and possibly mild long-term effect of the -10T allele on GLA expression might therefore manifest predominantly in the central and peripheral nervous system, which seems to be more susceptible to differences in GLA expression. Lately, other non-classical FD mutations have been described, resulting also in predominantly neurological manifestations [36,37]. Furthermore, our observations suggest that the observed neurological phenotype might be the result of GLA expression dysregulation in the presence of the -10T allele and an additional yet unknown neurological factor. This would explain Fabry-like disease manifestations even if GLA enzyme activities are measured within the low normal range. Life-time exposure to dysregulated GLA expression may thus lead to neurological manifestations.

A clinical approach with ERT in the index patient (agalsidase-beta, 1.0 mg/kg BW i.v. every other week, Fabrazyme, Genzyme) was started in 2006 and cardiac, renal and neurological examinations as well as biochemical analyses were performed in yearly follow-ups. ERT led to clinical stabilization of the FD manifestations, significant reduction of neuropathic pain, increase of daily activity and quality of life. Of note, within the first year of therapy the index patient showed significant pain reduction under ERT with standard dose of agalsidase-beta (1.0 mg/kg BW i.v. every other week, Fabrazyme, Genzyme). General disease stabilization and increased physical activity was also observed. During the worldwide agalsidase-beta shortage and subsequently dose reduction (0.5 mg/kg BW i.v. every other week, Fabrazyme, Genzyme) pain increased and daily physical activity was dramatically reduced due to physical weakness. This observation is underlined by Weidemann et al. [38] who reported a significant increase of pain attacks and crisis under enzyme reduction in a multi-center study for FD patients. The latter symptoms were completely restored in the index patient some months after re-switch to a regular dose of agalsidase-beta.

With respect to our findings, we suggest that patients carrying the -10T haplotype suffering from typical neuropathic pain and after exclusion of concurrent etiologies should be treated with established symptomatic medication (e.g. anticonvulsants blocking calcium channels, anticonvulsants and local anesthetics blocking sodium channels, opioids, etc.). In patients with therapy-resistant neuropathic pain ERT could be an option as shown in our index patient. Additionally, WML are no primary indication for ERT, but progression of lesion load should be discussed as ERT-indication, since ERT has been shown to ameliorate endothelial dysfunction in Fabry patients [39–42].

Of note, the frequency of the -10T allele is reported to be 7% within Caucasian populations [43]. Between 07/2011 and 12/2013, 49 patients, which were diagnosed as classical Fabry patients by genetic testing, presented at the Fabry center in Muenster. In the same period, 12 symptomatic patients were identified as -10T allele carriers without any further FD-causing mutation. This observation indicates an estimated incidence of ~1:11,500 symptomatic -10T allele carriers among all -10T allele carriers.

## Conclusions

Based on the presented complementary approach of clinical data and functional testing, we conclude that in patients with Fabry-typical neurological manifestations without coding *GLA* variants, the -10C>T substitution could be causal. A limitation of this study is the small number of patients and the retrospective design. To evaluate a pathogenic effect of the -10T allele and to clarify whether patients with the -10T allele and a typical clinical picture of FD might benefit from ERT for endorgan damage prevention, prospective studies with larger study populations are needed.

## Additional file

Additional file 1: Table S1. Patients’ characteristics. **Table S2.** Sequences and positions of oligonucleotides.

## Abbreviations

BW: Body weight
CDT: Cold detection threshold
ChIP: Chromatin immunoprecipitation
CPT: Cold pain threshold
CSF: Cerebrospinal fluid
DMA: Dynamic mechanical allodynia
EMSA: Electrophoretic mobility shift assay
ERT: Enzyme replacement therapy
FD: Fabry disease
FLAIR: Fluid attenuated inversion recovery
Gb3: Globotriaosylceramide
GLA: Alpha-galactosidase A
HPT: Heat pain threshold
i.v.: Intravenous
IENFD: Intra-epidermal nerve fiber density
IHKE: Immortalized human kidney epithelial cells
IVS: Intervening sequence
lyso-Gb3: Globotriaosylsphingosine
MDT: Mechanical detection threshold
MPT: Mechanical pain threshold
MRI: Magnetic resonance imaging
MPS: Stimulus–response-functions for pinprick
NRS: Numeric rating scale
PHS: Presence of paradoxical heat sensations
PPT: Blunt pressure
QST: Quantitative sensory testing
SFN: Small fiber neuropathy
TFEB: Transcription factor EB
TIA: Transient ischemic attack
TSL: Thermal sensory limen
UTR: Untranslated region
VDT: Vibration detection threshold
WDT: Warm detection threshold
WML: White matter lesions
WUR: Wind-up ratio
