# Supplementary material for: Cryptogenic stroke and small fiber neuropathy of unknown etiology in patients with alpha-galactosidase A -10T genotype
Source: Orphanet J Rare Dis. 2014 Nov 26;9:178. doi: 10.1186/s13023-014-0178-5 (PMC4255940; doi:10.1186/s13023-014-0178-5)
Supplement: Additional file 1: Table S1. — Patients’ characteristics. Table S2. Sequences and positions of oligonucleotides. [file 13023_2014_178_MOESM1_ESM.doc]

| **Table S1: Patients characteristics.** | | | | | | | | |
| --- | --- | --- | --- | --- | --- | --- | --- | --- |
|  |  |  |  | **FD typical abnormalities** | | | | |
| **Patient #** | **Gender** | **Age**  **[years]** | **Genotype** | **Cardiac** | | **Renal** | | **General** |
| **-10T carriers with neurological manifestations** | | | | | | | | |
| 1* | f | 63 | -10T/X | left-ventricular hypertrophy, diastolic ventricular dysfunction | none | | tinnitus | |
| 2 | f | 43 | -10T/X | none | none | | none | |
| 3 | m | 12 | -10T/Y | none | none | | none | |
| 4 | m | 15 | -10T/Y | none | none | | hyperhidrosis | |
| 5 | f | 46 | -10T/X | none | tubular proteinuria | | none | |
| 6 | f | 53 | -10T/X | none | none | | angiokeratoma, lysosomal cellular inclusions | |
| 7 | f | 65 | -10T/X | none | micro-albuminuria | | hypohidrosis, angiokeratoma, lysosomal cellular inclusions | |
| 8 | f | 49 | -10T/X | none | none | | hyperhidrosis | |
| 9 | f | 55 | -10T/X | none | none | | angiokeratoma | |
| 10 | f | 54 | -10T/-10T | none | none | | hypohidrosis | |
| 11 | f | 72 | -10T/X | none | none | | none | |
| 12 | m | 52 | -10T/Y | none | none | | none | |
| 13 | m | 35 | -10T/Y | none | none | | none | |
| **-10T carriers without neurological manifestations** | | | | | | | | |
| 14 | m | 19 | -10T/Y | none | none | | none | |
| 15 | m | 33 | -10T/Y | ICD after ventricular fibrillation | none | | none | |

Renal parameters determined by: ultrasonography, albumin/creatinine ratio, determination of eGFR (CKD-EPI formula). Cardiac parameters determined by: echocardiography, electrocardiogram. f: female; m: male; ICD: implantable cardioverter-defibrillator; FD: Fabry disease.*index patient

**Table S2:** **Sequences and positions of oligonucleotides.**

| **Oligonucleotides** | | **Sequence 5´-3´** | **Assignment** | |
| --- | --- | --- | --- | --- |
| **Oligonucleotide sequences for generation of serial promoter deletion constructs** | | | | |
| GLA_ss1 | | CTACTGTCATGGAAAAATGCTCG | -4011 | |
| GLA_ss2 | | GACTAATCTGCCCAGGGATC | -3019 | |
| GLA_ss3 | | GGTGAAGTGGGCTTTTCGC | -1783 | |
| GLA_ss4 | | GATTTAACAAACATTTACTGATTGCC | -1396 | |
| GLA_ss5 | | GTGGGAACCAGGACTCTTTG | -1106 | |
| GLA_ss6 | | GCATTTGCCTAGATGTGACTC | -845 | |
| GLA_ss7 | | GATAGACCAGCACTCAGACC | -756 | |
| GLA_ss8 | | CACACCAACCTCTAACGATAC | -425 | |
| GLA_ss9 | | GTTGCCAGAGAAACAATAACGTC | -110 | |
| GLA_as0 | | CCATTGTCCAGTGCTCTAGC | +104 reverse primer | |
| **Oligonucleotide sequences for EMSA probes** | | | | |
| WT-probe_ss | | AAATTTATGCTGTCCGGTCACCGTGACA | EMSA probe C allele forward | |
| WT-probe_as | | TGTCACGGTGACCGGACAGCATAAATTT | EMSA probe C allele reverse | |
| C>T-probe_ss | | AAATTTATGCTGTCCGGTTACCGTGACA | EMSA probe T allele forward | |
| C>T-probe_as | | TGTCACGGTAACCGGACAGCATAAATTT | EMSA probe T allele reverse | |
| **Oligonucleotide sequences for exon trapping experiments** | | | | |
| IVS2_for | GTGGAATTCGCTGCTAGCTTTCTCCTCAG | | | pSPL3 IVS2_forward |
| IVS2_rev | GTGGGATCCGATTGACAACCTGGACTCCC | | | pSPL3 IVS2_reverse |
| IVS4_for | GTGGAATTCGCAGCTTTTAATTTTACTAAGACC | | | pSPL3 IVS4_forward |
| IVS4_rev | GTGGGATCCGAAATAATTCAAACAAGAGAGGAGG | | | pSPL3 IVS4_reverse |
| IVS6_for | GTGGAATTCCTTGGGCCTAAATCTATTTTTTTCC | | | pSPL3 IVS6_forward |
| IVS6_rev | GTGGGATCCCAGAGTAGCTGGGACCACAG | | | pSPL3 IVS6_reverse |
| IVS4+919_for | GTGGAATTCCTGTCCCTCAACACTGCAAG | | | pSPL3 IVS4+919_reverse |
| IVS4+919_rev | GTGGGATCCGAAATAATTCAAACAAGAGAGGAGG | | | pSPL3 IVS4+919_forward |
| SD6 | TCTGAGTCACCTGGACAACC | | | forward pSPL3 specific |
| SA2 | ATCTCAGTGGTATTTGTGAGC | | | reverse pSPL3 specific |
| **Oligonucleotide sequences for ChIP** | | | | |
| GLA_ss8 | | CACACCAACCTCTAACGATAC | ChIP PCR forward | |
| GLA_as3B | | CTCGGGATCACTAAGGTGC | ChIP PCR reverse | |
| **Oligonucleotide sequences for real-time PCR** | | | | |
| GAPDH_for | | CTGCACCACCAACTGCTTAGCAC | real-time PCR GAPDH forward | |
| GAPDH_rev | | GTGATGGCATGGACTGTGGTCATGAG | real-time PCR GAPDH reverse | |
| GLA_for | | ACCAGCTTAGACAGGGAGAC | real-time PCR GLA forward | |
| GLA_rev | | AAGAGCGAGGTCCACCAATC | real-time PCR GLA reverse | |

*GLA* reference accession #: 4296
